# Acupuncture combined with Tongxieyaofang for diarrhea-type irritable bowel syndrome: A protocol for meta-analysis

**DOI:** 10.1097/MD.0000000000023457

**Published:** 2020-11-25

**Authors:** Yi-Lin Li, Cheng-Jiao Yao, Rong Lei, Fengjiao Xie, Qin Xiong, Li-Hong Luo, Pei-Min Feng

**Affiliations:** a Hospital of Chengdu University of Traditional Chinese Medicine, Chengdu; b North Sichuan Medical College; c Department of Geriatrics of the Affiliated Hospital; d Department of Health Management Center of the Affiliated Hospital, North Sichuan Medical College, Nanchong, Sichuan, China.

**Keywords:** acupuncture, irritable bowel syndrome, meta-analysis, protocol, Tongxieyaofang

## Abstract

**Background:**

As a traditional Chinese medicine external treatment method, acupuncture is characterized by simple operation, significant treatment effect and few side effects. Tong-Xie-Yao-Fang (TXYF), a Chinese patent medicine, combined with acupuncture has been widely used on treating Diarrhea Predominant Irritable Bowel Syndrome (IBS-D). However, the efficacy and safety of TXYF combined with acupuncture for the treatment of IBS-D are unclear. This study aims to investigate verify the efficacy and safety of TXYF combined with acupuncture for IBS-D.

**Methods and analysis:**

Randomized controlled trials of TXYF combined with acupuncture for all IBS-D will be searched in PubMed, the Cochrane Library, Embase, Web of Science, the China National Knowledge Infrastructure, Wanfang Database, Chinese Science and Technology Periodical Database, and Chinese Biomedical Literature Database from inception to October 20, 2020. And Baidu Scholar, Google Scholar, International Clinical Trials Registry Platform, and Chinese Clinical Trials Registry will be searched to obtain more relevant studies comprehensively. The methodological qualities, including the risk of bias, will be evaluated using the Cochrane risk of bias assessment tool, while confidence in the cumulative evidence will be evaluated using the Grading of Recommendations Assessment, Development and Evaluation (GRADE) approach. Two researchers will perform data extraction and risk of bias assessment independently. Statistical analysis will be conducted in RevMan 5.3.

**Results:**

Based on the current evidence, the potential rank of the efficacy and safety of TXYF plus acupuncture for IBS-D will be assessed.

**Conclusion:**

The findings of the study will provide helpful evidence for the efficacy and safety of TXYF combined with acupuncture in the treatment of IBS-D, facilitating clinical practice and further scientific studies.

## 1 Introduction

Irritable bowel syndrome (IBS), a common functional gastrointestinal disorder, is characterized by recurrent abdominal pain or abdominal discomfort (the latter has been removed from the Rome IV criteria) and abnormal bowel habits.^[1]^ On the basis of Rome IV criteria, IBS is divided into 4 subtypes based on symptoms, including IBS with prominent diarrhea (IBS-D), IBS with constipation (IBS-C), IBS with mixed symptoms of diarrhea and constipation (IBS-M), and untyped IBS (IBS-U).^[1]^ IBS-D is highly prevalent.^[2–3]^ It causes low work productivity^[4–5]^ and low quality of life.^[6]^ However, the pathogenesis of IBS-D is not yet clear, and its etiology is complex and may be caused by a variety of factors including visceral allergies, inflammatory responses, heredity, gastrointestinal motility disorders, intestinal infections, and psychosocial factors.

Conventional pharmacotherapies (CP), such as antispasmodics, antidiarrheal agents, antidepressants, 5-hydroxytryptamine3 (5-HT3) receptor antagonists, probiotics, and antibiotics, cannot achieve satisfactory clinical efficacy, and some of them are even associated with the risk of incidence of cardiovascular events and ischemic colitis.^[7]^ Therefore, an increasing number of IBS patients have turned to alternative medicine, especially for traditional Chinese medicine (TCM), for symptom alleviation. TXYF was first described in “Danxis Mastery of Medicine” and includes pericarpium citri reticulatae, rhizoma atractylodis macrocephalae, radix paeoniae alba, and radixsileris. TXYF has been widely used for the clinical treatment of IBS-D.^[8–9]^ Animal studies showed its efficacy in relieving smooth muscle contraction and decreasing visceral hypersensitivity,^[10–12]^ and the efficacy may be related to the regulation of 5-hydroxytryptamine and substance P in colonic tissues.^[10]^ Yuan et al had demonstrated that TXYF could inhibit the colonic hypermotility through preventing the influx of extracellular Ca^2+^ into the isolated rat colonic smooth muscle cells.^[13]^ TXYF has been shown in a preliminary clinical study to demonstrate an antipain and antidiarrhea effect on IBS-D patients.^[14]^ Several studies have revealed that acupuncture can attenuate visceral hyperalgesia and alleviate symptoms of IBS-D.^[15–16]^ In recent research, there has been a growing interest in investigating the possible role of acupuncture in improving symptoms and quality of life in IBS patients.^[17]^ In Chinese medicine practice, acupuncture, and related therapies are frequently used in conjunction with Chinese herbal medicine, and this combined treatment is generally assumed to provide better treatment effects.^[18–19]^

Some previous reviews regarding TXYF^[20–21]^ or acupuncture^[22]^ alone have suggested that both TXYF and acupuncture have a beneficial effect on IBS-D symptoms. Nonetheless, no systematic review or meta-analysis is available at present to assess the effect of acupuncture combined with TXYF on treating IBS-D. Therefore, the aim of this meta-analysis of randomized controlled trails is to evaluate the efficacy of acupuncture combined with TXYF for IBS-D, results in this study might prove to be more reliable for clinical practice and decision-making.

## 2 Method

### 2.1 Protocol register

This protocol of systematic review and meta-analysis has been drafted under the guidance of the preferred reporting items for systematic reviews and meta-analyses protocols. Moreover, the protocol has been registered on the INPLASY website (registration number is INPLASY2020100052).

### 2.2 Ethics

Ethical approval is not required because there is no patient recruitment and personal information collection, and the data included in our study are derived from published literature.

### 2.3 Inclusion criteria for study selection

#### 2.3.1 Types of studies

Randomized controlled trials including TXYF combined with acupuncture for the treatment of IBS-D will be included. The language will be limited to Chinese and English.

#### 2.3.2 Types of participants

Patients who are diagnosed with IBS according to Manning or Rome I, II, III, or IV criteria will be included in the analysis, regardless of their age, gender, ethnicity, or background. Participants need to be able to participate in acupuncture combined with TXYF treatment to be eligible for inclusion.

#### 2.3.3 Types of interventions

The control group was treated with western medicine only, and western medicine type was not limited; the treatment group was treated with TXYF combined with acupuncture application. The duration of treatment in both groups was not limited.

#### 2.3.4 Types of outcome measures

##### 2.3.4.1 Primary outcomes

Adequate relief (AR) is used to evaluate the degree of alleviation of IBS symptoms, with relief of abdominal pain or abdominal discomfort as the criterion. During the treatment and follow-up, the patients evaluate themselves once every week. The primary endpoint is set at the end of the treatment (week 6), and the statistics are also performed during follow-ups (weeks 12 and 18) for efficacy duration assessment. “Yes” is defined as positive symptom improvements in more than 3 weeks during the 6-week period; “no” is defined as positive symptom improvements in less than 3 weeks.^[23]^ The indicator has been applied in several studies^[24–25]^ and was found to have significant correlations with intestinal function and quality-of-life (QOL).^[26]^

##### 2.3.4.2 Secondary outcomes

###### 2.3.4.2.1 IBS Symptom severity score

IBS symptom severity score (SSS)^[27]^ is used to evaluate the degree of severity of IBS-D in the patients. A total score of 500 is calculated from abdominal pain degree, abdominal pain frequency, abdominal distension degree, defecation satisfaction, and influence on life. If the score is lower than 75, the patient is considered to be in remission. The mild, moderate, and severe boundary values are 75–175, 175–300, and above 300, respectively. The effectiveness, reliability, and sensitivity of IBS SSS to treatment are verified.

###### 2.3.4.2.2 Bristol stool form scale

Grading standard in the Bristol stool form scale (BSS)^[28]^ is employed to record stool characteristics of the patients with IBS-D in this study. The scale is a visual descriptive figure reflecting gastrointestinal transit time. The total score of the scale ranges from 1 to 7 according to the 1–7 types (from constipation to diarrhea) of stool characteristics.

###### 2.3.4.2.3 Irritable bowel syndrome quality-of-life questionnaire

Irritable bowel syndrome quality-of-life questionnaire (IBS-QOL) is a specialized scale developed by Patrick et al^[29]^ for patients with IBS. It is composed of 34 items of self-assessment ranging from dysphoria, interference with activity, body image, health worry, food avoidance, social reaction, to sexual relationships, to assess QOL. The “patients” QOL is measured by a 5-point linear scale, and each question has a 5-point Likert scale (score 1–5). The total score of the scale ranges from 34 to 170. The scale achieves good measurement validity.

###### 2.3.4.2.4 Hospital anxiety and depression scale

Hospital anxiety and depression scale (HADS) is used to assess the level of anxiety and depression in patients with IBS-D.^[30]^ It comprises 14 questions, with 7 questions each on anxiety and depression. Each question has a 4-point Likert scale (score 0–3). The total score of the scale ranges from 0 to 42. A score greater than 8 in each subscale indicates anxiety/depression. IBS-SSS and BSS are completed before treatment (week 0), at the end of treatment (week 6), and during follow-ups (weeks 12 and 18). IBS-QOL and HADS are completed before treatment (week 0) and at the end of treatment (week 6).

### 2.4 Exclusion criteria

1. For repeated literatures, choosing the one with the most complete information;
2. Studies with missing data, and cannot get the data after contacting the author;
3. Literature with incorrect research data that cannot be obtained after contacting the author;
4. Papers assessed as high risk of bias by randomization or concealed distribution;
5. Papers whose intervention was combined with other traditional Chinese medicine therapies;
6. Papers with no relevant outcome indicators.

### 2.5 Search strategy

PubMed, the Cochrane Library, Embase, Web of Science, the China National Knowledge Infrastructure, Wanfang Database, Chinese Science and Technology Periodical Database, and Chinese Biomedical Literature Database were searched by computer to collect randomized controlled trials of TXYF combined with acupuncture in the treatment of IBS-D. And the retrieval time was from the establishment of each database to Oct 20, 2020. At the same time, search Baidu, Google Scholar, International Clinical Trials Registry Platform, and Chinese Clinical Trials Registry to get more comprehensive data. Keywords were: “Irritable bowel syndrome,” “acupuncture,” “Tongxieyaofang,” and so on. PubMed retrieval strategies are shown in Table 1.

**Table 1:** Search strategy used in PubMed database.

Number	Search terms
1	Irritable Bowel Syndromes
2	Syndrome, Irritable Bowel
3	Syndromes, Irritable Bowel
4	Colon, Irritable
5	Irritable Colon
6	Colitis, Mucous
7	Colitides, Mucous
8	Mucous Colitides
9	Mucous Colitis
10	1 or 2–9
11	Pharmacopuncture
12	acupuncture
13	11 or 12
14	Tong Xie Yao Fang
15	TXYF formula
16	TXYF
17	14 or 15–16
18	Randomized Controlled Trials
19	Randomized Controlled Trial
20	RCT
21	controll clinical trial
22	Trials
23	Clinical Trials, Randomized
24	Trials, Randomized Clinical
25	Controlled Clinical Trials, Randomized
26	18 or 19–25
27	10 and 17 and 26

### 2.6 Data extraction

Records from database searches will be managed by EndNoteX7 software. The titles and abstracts of each record retrieved will be checked by 2 independent authors according to eligibility criteria. The full texts of potentially relevant studies will be retrieved for further assessment. Disagreements will be resolved by discussion or consultation of a 3rd author. A data spreadsheet will be created using Microsoft Excel 2019 to collect relevant information and data. The information, including author, year of publication, sample size, intervention, and outcome will be extracted from each study and entered into the spreadsheet.

**Figure 1:**
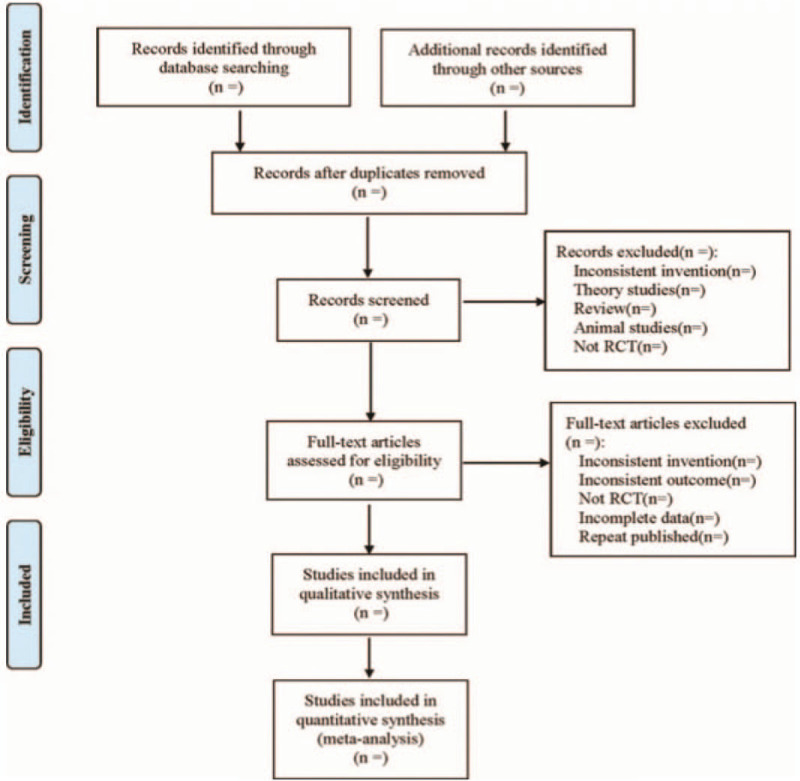
Flow diagram.

The extraction contents were as follows: ①Included basic research information (study title, first author, publication time, sample size, sex ratio, average age, average course of disease, etc.); ②Information about intervention measures (western medicine used in the control group, its dose, name, course of treatment; the acupoints of acupuncture, treatment frequency, and course of treatment in the treatment group; the ingredients, doses, frequency, treatment course of TXYF in the treatment group); ③Risk evaluation items of bias in randomized controlled trials; ④The outcomes and relevant measurement data. The literature screening process is shown (Fig. 1. Flow diagram).

### 2.7 Risk of bias assessment

Two researchers independently evaluated the risk of bias in randomized controlled trials in accordance with the Cochrane Handbook of Systematic Reviewers, including the following items: random sequence generation, allocation concealment, blinding of participants and personnel, blinding of outcome assessment, incomplete outcome data, selective reporting, and other bias. The quality of studies was classified as being at of high, unclear, or low risk of bias. After completion, they would recheck. In case of a disagreement, they would discuss. If no agreement could be reached, a decision would be made in consultation with researchers from the third party.

### 2.8 Statistical analysis

#### 2.8.1 Data synthesis

The RevMan 5.3 software provided by the Cochrane Collaboration was used for statistical analysis. ①Combined effect amount: Relative risk was selected as the statistic for the dichotomous variable. For continuous variables, Weighted Mean Difference was selected when the tools and units of measurement indicators are the same, Standardized Mean Difference was selected with different tools or units of measurement, and all the above were represented by effect value and 95% confidence interval (CI). ②Heterogeneity test: Q test was used to qualitatively determine inter-study heterogeneity. If *P* ≥ .1, there was no inter-study heterogeneity, If *P* < .1, it indicated inter-study heterogeneity. At the same time, *I*^2^ value was used to quantitatively evaluate the inter-study heterogeneity. If *I*^2^ ≤ 50%, the heterogeneity was considered to be good, and the fixed-effect model was adopted. If *I*^2^ > 50%, it was considered to have significant heterogeneity, the source of heterogeneity would be explored through subgroup analysis or sensitivity analysis. If there was no obvious clinical or methodological heterogeneity, it would be considered as statistical heterogeneity, and the random effect model would be used for analysis. Descriptive analysis was used if there was significant clinical heterogeneity between the 2 groups and subgroup analysis was not available.

#### 2.8.2 Dealing with missing data

When occurring missing, incomplete, and unclear data, we will contact the primary author to obtain it. If data are not acquired, we will analyze the present data and discuss its possible effect on the conclusion.

#### 2.8.3 Heterogeneity and subgroup analysis

Subgroup analysis will also be employed to explore the possible causes of heterogeneity. Subgroup analysis will be based on possible factors that may lead to heterogeneity, such as intervention measures, control measures, length of treatment, or quality of articles, etc. If quantitative synthesis not appropriate, we will conduct a narrative synthesis.

#### 2.8.4 Sensitivity analysis

In order to test the stability of meta-analysis results of indicators, a one-by-one elimination method will be adopted for sensitivity analysis.

#### 2.8.5 Reporting bias

For the major outcome indicators, if the included study was ≥10, funnel plot was used to qualitatively detect publication bias. Moreover, Egger and Begg test are used to quantitatively assess potential publication bias.

#### 2.8.6 Evidence quality evaluation

The evidence evaluation of results is summarized by the Grading of Recommendations Assessment, Development and Evaluation (GRADE) system. It contains 5 domains (bias risk, consistency, directness, precision, and publication bias). And the quality of evidence will be rated as high, moderate, low, and very low.

## 3 Discussion

IBS is a common disease, but no universally accepted therapy is available at present to halt its progression. Hence, increasing patients and practitioners have turned to acupuncture combined with TCM for treatment. TXYF, a prescription in TCM that has been extensively applied in relieving IBS-associated symptoms since the Yuan Dynasty,^[31]^ is comprised of Atractylodes rhizome, white peony root, dried old orange peel, and ledebouriella root. TXYF has been shown in a preliminary clinical study to demonstrate an antipain and antidiarrhea effect on IBS-D patients^[15]^; Besides, it can also regulate cytokine levels in the colonic mucosa, which may account for a potential molecular mechanism of its effect on IBS-D.^[32]^ In Chinese medicine practice, acupuncture and related therapies are frequently used in conjunction with Chinese herbal medicine, and this combined treatment is generally assumed to provide better treatment effects.^[19,20]^ Several studies have revealed that acupuncture can attenuate visceral hyperalgesia and alleviate symptoms of IBS-D^[16,17]^.

However, so far, there is no systematic review and meta-analysis assessing TXYF combined with acupuncture for treating IBS-D. This is the first protocol for systematic review and meta-analysis evaluating the efficacy and safety of TXYF combined with acupuncture for the treatment of IBS-D. This systematic evaluation and meta-analysis can provide evidence-based evidence for clinicians to use TXYF combined with acupuncture for the treatment of IBS-D. However, the study has some limitations. Due to different types of acupuncture and different times of application, the results were affected and the bias was caused. And only studies published in English and Chinese are retrieved, so important studies published in other languages may be missed.

## Author contributions

**Conceptualization:** Peimin Feng.

**Data curation:** Rong Lei.

**Formal analysis:** Cheng-jiao Yao.

**Funding acquisition:** peimin feng.

**Methodology:** Cheng-jiao Yao.

**Project administration:** Rong Lei.

**Software:** Cheng-jiao Yao.

**Supervision:** Fengjiao Xie, Qin Xiong, Li-hong Luo.

**Visualization:** Fengjiao Xie, Qin Xiong, Li-hong Luo.

**Writing – original draft:** Yi-lin Li.

**Writing – review & editing:** Yi-lin Li.
